# Essential Role of WetA, but No Role of VosA, in Asexual Development, Conidial Maturation and Insect Pathogenicity of Metarhizium *robertsii*

**DOI:** 10.1128/spectrum.00070-23

**Published:** 2023-03-14

**Authors:** Jin-Guan Zhang, Ke Zhang, Si-Yuan Xu, Sheng-Hua Ying, Ming-Guang Feng

**Affiliations:** a MOE Laboratory of Biosystems Homeostasis & Protection, College of Life Sciences, Zhejiang University, Hangzhou, Zhejiang, China; University of Natural Resources and Life Sciences Vienna

**Keywords:** asexual developmental activator, conidial maturation, entomopathogenic fungi, spore wall assembly

## Abstract

Conidial maturation, which is crucial for conidial quality, is controlled by the asexual development activator WetA and the downstream, velvety protein VosA in Aspergillus. Their orthologs have proved functional in conidial quality control of Beauveria bassiana, as seen in Aspergillus, but are functionally unexplored, in Metarhizium *robertsii*, another hypocrealean insect pathogen. Here, WetA and VosA prove essential and nonessential for *M. robertsii*'s life cycle, respectively. Disruption of *wetA* increased hyphal sensitivity to oxidative stress and Congo red-induced cell wall stress, but had little impact on radial growth. The Δ*wetA* mutant was severely compromised in conidiation capacity and conidial quality, which was featured by slower germination, decreased UV resistance, reduced hydrophobicity, and deformed hydrophobin rodlet bundles that were assembled onto conidial coat. The mutant's virulence was greatly attenuated via normal infection due to a blockage of infection-required cellular processes. All examined phenotypes were unaffected for the Δ*vosA* mutant. Intriguingly, mannitol was much less accumulated in the 7- and 15-day-old cultures of Δ*wetA* and Δ*vosA* than of control strains, while accumulated trehalose was not detectable at all, revealing little a link of intracellular polyol accumulation to conidial maturation. Transcriptomic analysis revealed differential regulation of 160 genes (up/down ratio: 72:88) in Δ*wetA.* These genes were mostly involved in cellular component, biological process, and molecular function but rarely associated with asexual development. Conclusively, WetA plays a relatively conserved role in *M. robertsii*’s spore surface structure, and also a differentiated role in some other cellular processes associated with conidial maturation. VosA is functionally redundant in *M. robertsii* unlike its ortholog in *B.* bassiana.

**IMPORTANCE** WetA and VosA regulate conidiation and conidial maturation required for the life cycle of Beauveria bassiana, like they do in Aspergillus, but remain functionally unexplored in Metarhizium *robertsii*, another hypocrealean pathogen considered to have evolved insect pathogenicity ~130 million years later than *B.* bassiana. This study reveals a similar role of WetA ortholog in asexual development, conidial maturation, and insect pathogenicity, and also its distinctive role in mediating some other conidial maturation-related cellular events, but has functional redundancy of VosA in *M. robertsii*. The maturation process vital for conidial quality proves dependent on a role of WetA in spore wall assembly but is independent of its role in intracellular polyol accumulation. Transcriptomic analysis reveals a link of WetA to 160 genes involved in cellular component, biological process, and molecular function. Our study unveils that *M. robertsii* WetA or VosA is functionally differential or different from those learned in *B.* bassiana and other ascomycetes.

## INTRODUCTION

Hypocrealean insect pathogens are well represented by *Beauveria* (Cordycipitaceae) and Metarhizium (Clavicipitaceae) that undergo saprophytic, insect-pathogenic, and plant-endophytic lifestyles (1–3). Their insect pathogenicity is considered to have evolved from plant endophytes or pathogens around the Triassic-Jurassic boundary 200 million years (MY) ago, and Metarhizium could have appeared 130 MY later than *Beauveria* on earth (4–7). Perhaps due to this 130-MY difference, the 2 fungal lineages have differential, or even different, gene expression networks that control cellular processes that are vital for their adaptation to insect-pathogenic lifestyle and environment (8–10). As examples, appressoria as infective structures of plant-pathogenic fungi are often formed by Metarhizium spp. during host infection (11–13) but are rarely observed in Beauveria bassiana (14). Msn2, a core transcription factor downstream of the mitogen-activated protein kinase (MAPK) Hog1 cascade, differentially regulate 12% more and 3% fewer genes in the genome of Metarhizium *robertsii* than of *B.* bassiana under heat shock and oxidative stress, respectively (15). A small cysteine-free protein (CFP), as a high virulence factor, differentially regulates 1,818 genes in *B.* bassiana (16), whereas only 604 genes are differentially regulated by its homolog, also proven as a virulence factor in *M. robertsii* (17). Therefore, *B.* bassiana and *M. robertsii* have become model species for in-depth insight into evolutionary trajectory of fungal adaptation to an insect-pathogenic lifestyle.

Asexual spores (conidia) crucial for insect-pathogenic lifecycles can, usually, be massively produced as active ingredients of fungal pesticides on artificial substrata. Metarhizium produces chained conidia on phialides like Aspergillus and *Penecillium* (18, 19), while *Beauveria* starts conidiation through the formation of spore balls, which comprise tiny clustered zigzag rachises (conidiophores) and conidia produced on the rachises. Upon formation, spore balls increase steadily in size and density over the time of incubation, and are eventually scattered into a layer of conidial powder on substratum at the time of maturation (20). Despite different conidiation modes, *B.* bassiana and *M. robertsii* have sets of genes orthologous to those acting as components of central developmental pathway ([CDP]; *brlA*, *abaA*, and *wetA*) to mediate conidiophore development and conidiation (21–24) and of upstream developmental activation pathway (*fluG* and *flbA*–*flbE*) to activate the expression of the key CDP gene *brlA* in Aspergillus nidulans (24–33). Recent studies have confirmed that *brlA* and *abaA* regulate asexual development in *B.* bassiana (20) and *M. robertsii* (34), as they do in A. nidulans. The regulated processes include aerial conidiation and submerged blastospore production mimicking proliferation *in vivo* after hyphal invasion into insect hemocoel. However, gene expression networks regulated by either *brlA* or *abaA* are much simpler in *M. robertsii* than in *B.* bassiana, despite similar sizes of their genomes (4, 6). Knockout mutations of *brlA* and Δ*abaA* resulted in differential regulation of 255 and 233 genes in *M. robertsii* (34), but 1,513 and 2,869 genes in *B.* bassiana (33), respectively. The homologs of *fluG* and 5 fluffy genes (*flbA*–*flbE*) as activators of the *brlA* expression in A. nidulans have proven essential for insect pathogenicity, virulence, and stress tolerance, but nonessential for the *brlA* activation in *B.* bassiana (35–37). These studies indicate conserved roles for *brlA* and *abaA* in asexual developmental control of hypocrealean insect pathogens, but a distinct scenario for the activation of *brlA* in *B.* bassiana.

The maturation process of conidia forming on phialides or zigzag rachises determines conidial quality vital for insecticidal activity, and is accompanied by an assembly of cell wall components and an accumulation of intracellular polyols, such as trehalose and mannitol, which are crucial for stress tolerance (38–40). In A. nidulans, conidial maturation is controlled by the third CDP gene *wetA* and the downstream velvety protein gene *vosA* (18, 19). WetA was first proposed to activate a set of genes involved in spore-specific functions (24), and has been tied to several global regulators involved in conidiation and secondary metabolism (41, 42). The downstream VosA interacts with the other velvety protein VelB to form a VelB-VosA hetero-dimer that functions in trehalose biogenesis and spore wall completion (43, 44). In recent transcriptome analysis, WetA, VosA, and VelB have been shown to mediate sets of genes associated with heterotrimeric G-protein signal transduction, MAPK cascades, spore wall formation and structural integrity, and asexual development, and play interdependent, overlapping or distinct roles in morphological development and metabolic remodeling in A. nidulans (45). In Fusarium graminearum, WetA was characterized as the production of longer conidia with fewer septa, the elevation of conidial sensitivity to oxidative and heat stresses, and the reduced survival rate of aged conidia in the absence of its coding gene (46). Likewise, abnormal conidia showing loose cell walls, delayed germination, and altered sensitivities to stress cues were observed in the Δ*wetA* mutants of *Penicillium* spp. (47, 48). Exceptionally, deletion of *wetA* in Monascus ruber had no effect on conidial morphology, size, number, structure, and germination (49). The previous studies have revealed an array of important, but differential, cellular processes associated with conidial maturation in different ascomycetes. The role of VosA in conidial maturation remains unclear in the fungi other than Aspergillus.

The *wetA* and *vosA* orthologs in fungal insect pathogens have been functionally elucidated only in *B.* bassiana, in which *wetA* plays a greater role than *vosA* in sustaining conidial yield, morphology, size and density, hydrophobicity, cell wall integrity, and virulence but *vice versa* in trehalose accumulation into mature conidia (50). The different conidiation modes of *M. robertsii* and *B.* bassiana implicate that their conidial maturation may involve certain subtly different cellular events. This study seeks to characterize functions of *wetA* and *vosA* in *M. robertsii*. Our emphasis is placed upon the effects of targeted gene disruptions on asexual development, conidial maturation, stress tolerance, and insect pathogenicity. Unveiling the roles of *wetA* and *vosA* in *M. robertsii* will deepen an insight into the genetic control of conidial maturation in insect-pathogenic Hypocreales, and help to improve large-scale production technology and management of high-quality conidia as active ingredients of fungal pesticides.

## RESULTS

### Domain architecture of WetA and VosA in *M. robertsii*.

**FIG 1:**
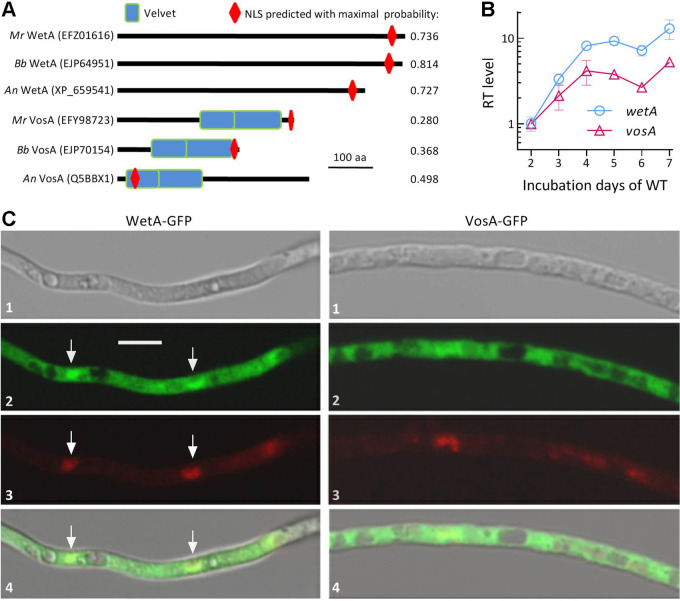
Domain architectures, transcriptional profiles, and subcellular localization of fungal WetA and VosA orthologs. (A) Comparative domain architectures of WetA and VosA orthologs in A. nidulans (*An*), *B.* bassiana (*Bb*) and *M. robertsii* (*M*_r_). (B) Relative transcript (RT) levels of *wetA* and *vosA* in the *M*_r_ WT strain during a 7-day incubation on PDA at the optimal regime of 25°C and 12:12 (L:D) with respect to a standard on day 2. Error bars: standard deviations (SDs) of the means from 3 independent cDNA samples analyzed via qPCR. (C) Laser scanning confocal microscopic (LSCM) images (scale: 5 μm) for subcellular localization of WetA-GFP and VosA-GFP fusion proteins expressed in the hyphae of *M*_r_ WT. Arrows indicate nuclei. Rows 1 to 4 are bright, expressed, DAPI-stained, and merged views of the same microscopic field, respectively.

Orthologous WetA and VosA were located in *M. robertsii* genome (4) via BLASTp search with the query sequences of WetA and VosA in A. nidulans (XP_659541, 555 amino acids [aa]; Q5BBX1, 430 aa) or *B.* bassiana (EJP64951, 639 aa; EJP70154, 273 aa). The located WetA (EFZ01616, 645 aa) share higher sequence identity with the query of A. nidulans (71.2% with e-value 7 × 10^−8^, total score 53.9 and coverage 10%) than of *B.* bassiana (56.6% with e-value 1 × 10^−28^, total score 215 and coverage 90%). The located VosA sequence (EFY98723, 397 aa) is more identical to the query of *B.* bassiana (77.04% with e-value 1 × 10^−111^, total score 326 and coverage 71%) than of A. nidulans (35.71% with e-value 4 × 10^−28^, total score 99.4 and coverage 35.71%). Conventional (SMART or NCBI) domain analysis revealed a lack of any known domain in WetA orthologs (Fig. 1A), although A. nidulans WetA was predicted to act as a DNA-binding factor at http://www.pantherdb.org/ and take part in asexual spore wall assembly, conidium formation, and pigment biosynthesis. Orthologous VosA features 2 partially overlapping Velvety domains at C-terminus in *B.* bassiana or *M. robertsii* but at N-terminus in A. nidulans. A nuclear localization signal (NLS) motif was predicted from the C termini of 3 WetA orthologs at high probabilities of 0.727 to 0.814 and the VosA C termini of *B.* bassiana and *M. robertsii* at lower probabilities (≤0.368). The N-terminal NLS motif of A. nidulans VosA was predicted a probability of 0.498. Obviously, either WetA or VosA orthologs in the mentioned fungi are similar in domain architecture but different in molecular size. In phylogeny, WetA and VosA orthologs found in the genomes of selected ascomycetes are clustered to the clades of their host lineages (Fig. S1).

### Transcriptional profiles and subcellular localization of WetA and VosA.

Both *wetA* and *vosA* were expressed in the wild-type strain *M. robertsii* ARSEF 2575 (WT hereafter) during a 7-day incubation on potato dextrose agar (PDA) at the optimal regime of 25°C and 12 h/12 h light/dark photoperiod (Fig. 1B); *wetA* was consistently expressed at higher level than *vosA*, despite their similar upregulation trends over the time of incubation. Green fluorescence-tagged WetA fusion protein (WetA-GFP) expressed in WT were localized in both nuclei and cytoplasm of hyphal cells, but accumulated more in the nuclei (Fig. 1C). The VosA-GFP fusion protein was more evenly distributed in the nuclei and cytoplasm. These observations are in accordance with the NLS motif of WetA predicted at much higher probability than of VosA in *M. robertsii*.

### Roles of WetA and VosA in radial growth and stress tolerance.

**FIG 2:**
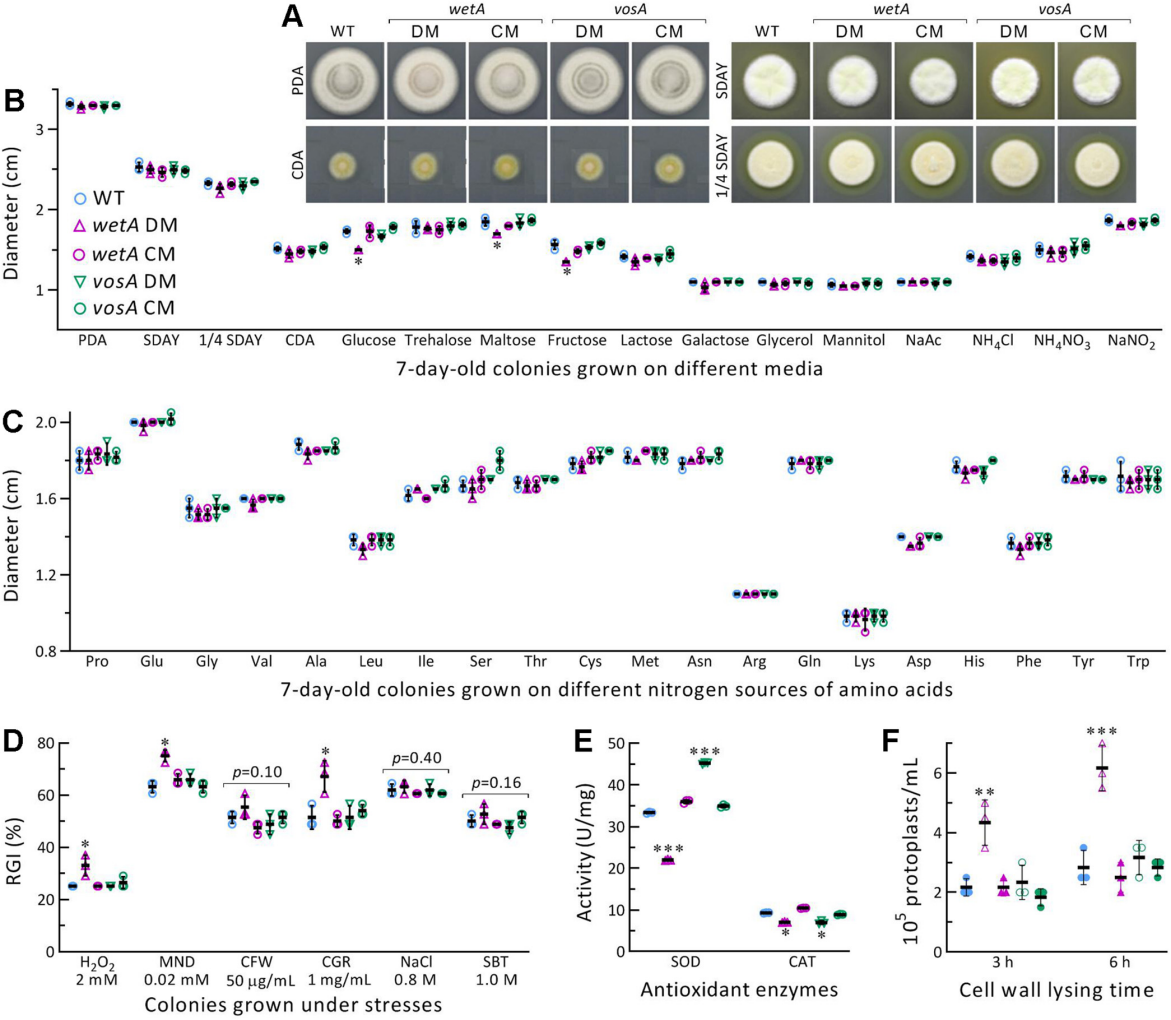
Roles of WetA and VosA in radial growth and stress response of *M. robertsii*. (A) Images for fungal colonies (DM, deletion mutant; CM, complemented mutant; WT, wild-type) incubated on rich (PDA, SDAY, and 1/4 SDAY) and minimal (CDA) media for 7 days at the optimal regime of 25°C and 12:12 (L:D). (B and C) Diameters of fungal colonies incubated at the optimal regime for 7 days on rich media, minimal CDA, and CDAs amended with different carbon or nitrogen sources. (D) Relative growth inhibition (RGI) percentages of fungal colonies grown at 25°C for 7 days on CDA plates supplemented with indicated concentrations of chemical stressors (SBT, sorbitol; MND, menadione; CGR, Congo red; CFW, calcofluor white). (E) Total SOD and catalase (CAT) activities quantified in the protein extracts of 3-day-old PDA cultures. (F) Concentration of protoplasts released from cells after 3 and 6 h of cell wall lysing in 1 M NaCl containing snailase and lysing enzymes of 10 mg/mL at 37°C. *, *P < *0.05 or ***, 0.001 in Tukey's test. Error bars: SDs of the means from three independent replicates.

The disruption mutants (DM) and complement mutants (CM) of *wetA* and *vosA* generated in the WT background (Fig. S2 and Table S1) (detailed in Methods) were incubated 7 days on different media at the optimal regime after initiation of radial growth with ~10^3^ conidia (in 1 μL of a 10^6^ conidia/mL suspension). Unlike fluffy colony morphology in *B.* bassiana (50), the DM strains displayed invisible defects in radial growth on the rich media PDA, Sabouraud dextrose agar plus yeast extract (SDAY) and ¼ SDAY (amended with ¼ of each SDAY nutrient) or the minimal Czapek-Dox agar (CDA) (Fig. 2A). The diameters of almost all 7-day-old colonies differed insignificantly between the DM strains and their control (WT and CM) strains (*P > *0.05 in Tukey’s test) on the rich media or the minimal CDA and CDAs amended with different carbon or inorganic nitrogen sources (Fig. 2B) or with organic nitrogen sources of different amino acids (Fig. 2C). The *wetA* DM showed significant, but moderate, growth defect (*P < *0.05 in Tukey’s test) only on the carbon sources of glucose, fructose, and maltose.

Stress assays on CDA supplemented with chemicals were conducted to reveal whether *wetA* and *vosA* were involved in some stress responses of *M. robertsii*, as seen in *B.* bassiana (50). Only the Δ*wetA* mutant was significantly more sensitive than the control strains to oxidative stress induced by H_2_O_2_ (2 mM) or menadione (0.02 mM), and cell wall stress induced by Congo red (1 mg/mL), while the tested strains were equally responsive to either the other cell wall antagonist calcofluor white (50 μg/mL) or osmotic stress induced by NaCl (0.8 M) or sorbitol (1 M) (Fig. 2D). In Δ*wetA*, increased sensitivity to oxidative stress correlated with reduced activities of superoxide dismutases (SOD) and catalases (Fig. 2E), which scavenge intracellular superoxide anions and H_2_O_2_, respectively (51). Interestingly, the Δ*vosA* mutant without antioxidant response showed increased SOD activity and decreased catalase activity. Additionally, impaired cell wall integrity, suggested by increased Δ*wetA* sensitivity to Congo red, was further shown with significantly more release of protoplasts from hyphal cells of Δ*wetA* than of the remaining strains after 3 or 6 h of cell wall lysing (Fig. 2F).

These data demonstrated significant role of *wetA* in *M. robertsii*'s responses to 2 oxidants and cell wall perturbing Congo red, despite its dispensability for radial growth under normal culture conditions and response to osmotic agents, and the other cell wall antagonist. There was no role for *vosA* in all tested phenotypes except for the counteracting effect on SOD and catalase activities.

### Essential versus null role of WetA versus VosA in asexual development and conidial quality control.

**FIG 3:**
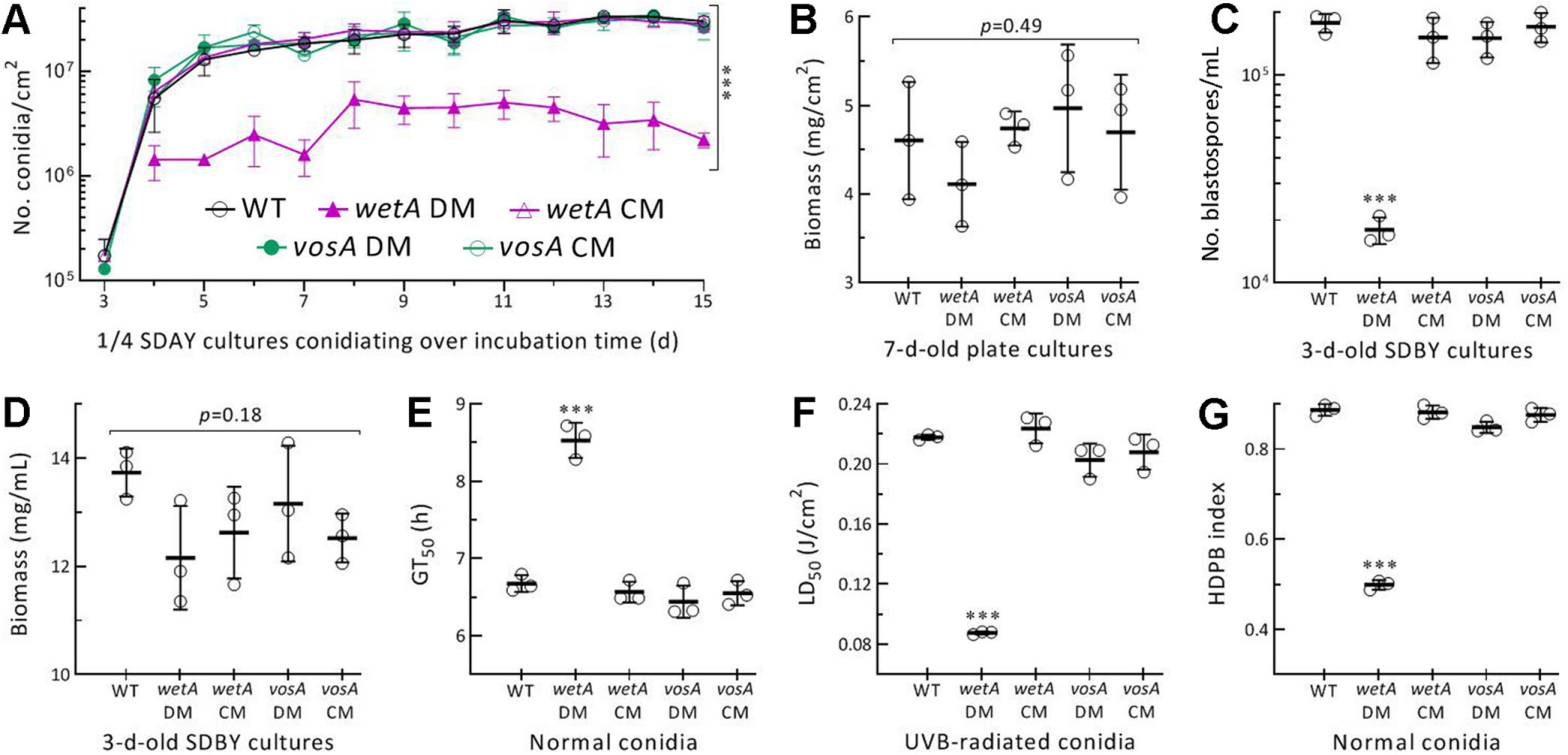
Essential versus null role of WetA versus VosA in asexual development and conidial quality control of *M. robertsii*. (A and B) Conidial yields assessed from the cultures of tested strains (DM, deletion mutant; CM, complemented mutant; WT, wild-type) during a 15-day incubation on 1/4 SDAY at 25°C and L:D 12:12, and biomass levels quantified from the 7-day-old cultures. All cultures were initiated by spreading 100 μL of a 10^7^ conidia/mL suspension per plate. (C and D) Blastospore yields and biomass levels assessed from the 3-day-old liquid cultures prepared by shaking a 10^6^ conidia/mL suspension in SDBY at 25°C. (E to G) Indices for viability (GT_50_ at 25°C), hydrophobicity (HDPB) (assessed in an aqueous-organic system) and UVB resistance of conidia collected from the 15-day-old cultures. *, *P < *0.05; **, 0.01; or ***, 0.001 in Tukey's test. Error bars: SDs of the means from 3 independent replicates.

Conidial yields were assessed daily from the cultures of all tested strains during a 15-day incubation at the optimal regime after 100 μL suspension (10^7^ conidia/mL) was spread per plate of 1/4 SDAY for culture initiation. The Δ*wetA* mutant showed a 1 -day delay in conidiation, and a conidial yield was reduced by 72% to 92% in the 4- to 15-day-old cultures, whereas little change in conidial yield was observed in Δ*vosA* (Fig. 3A). Biomass levels assessed from the 7-day-old plate cultures showed no variability (*P = *0.49) among the DM and control strains (Fig. 3B), suggesting no link of the Δ*wetA* mutant's conidiation defect to hyphal growth. Submerged blastospore production in 3-day-old SDBY (agar-free SDAY) cultures was reduced by 90% in Δ*wetA* but not affected in Δ*vosA* relative to the control strains (Fig. 3C), accompanied by an insignificant biomass variation (*P = *0.18) in the same cultures of all tested strains (Fig. 3D). These data indicated an essentiality of *wetA*, but a dispensability of *vosA*, for aerial conidiation and submerged blastospore production.

As an index of conidial viability, median germination time (GT_50_) at 25°C was significantly prolonged by 28% in Δ*wetA* versus WT (Fig. 3E), accompanied by a 60% reduction in median lethal dose (LD_50_) of UVB irradiation (Fig. 3F). Conidial hydrophobicity required for adhesion to insect cuticle (14) was largely lowered by 44% in Δ*wetA* (Fig. 3G). However, none of the properties were significantly affected in Δ*vosA* compared to the control strains, suggesting no role for *vosA* in conidial quality control of *M. robertsii*.

**FIG 4:**
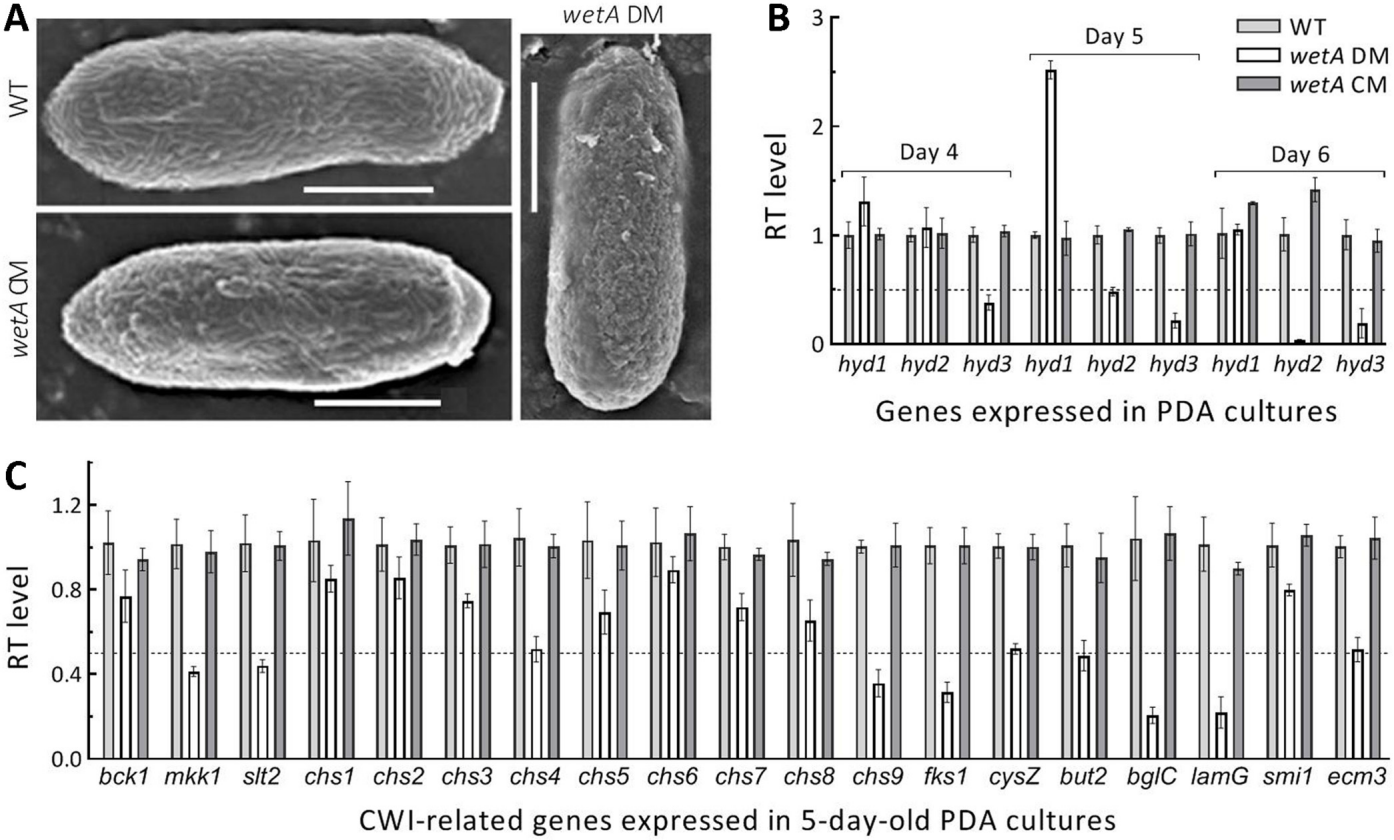
Impact of *wetA* disruption on microstructures of conidial surfaces and expression of genes involved in cell wall integrity (CWI). (A) SEM images for microstructures of conidial surfaces. Note the *wetA* DM's impaired and deformed hydrophobin rodlet bundles. (B and C) Relative transcript (RT) levels of 3 hydrophobin genes (*hyd1* to 3) and 19 CWI-related genes in the PDA cultures of *wetA* mutants with respect to the WT strain. The dashed line indicates a significance of 1-fold downregulation. Error bars: SDs of the means from 3 independent replicates.

Conidial hydrophobicity reduced in Δ*wetA* was further revealed with scanning electron microscopic (SEM) images of conidial surfaces. Hydrophobin rodlet bundles were well defined and intact on the conidial surfaces of the control strains, but impaired or deformed on the conidial surfaces of Δ*wetA* (Fig. 4A), indicating, again, an impairment of the mutant's cell wall integrity. For further insight into the impaired spore wall, expression levels of 3 hydrophobin genes (*hyd1* to 3) found in the fungal genome were assessed via real-time quantitative PCR (qPCR) with paired primers (Table S2). As a result, *hyd2* and *hyd3* were sharply downregulated in the 5- and 6-day-old cultures of Δ*wetA* versus the control strains (Fig. 4B). In addition, half of 19 other genes involved in cell wall composition and integrity were also downregulated in the mutant's 5-day-old cultures (Fig. 4C). The repressed genes encode the MAPK Slt2 and the MAPK kinase Mkk1 required for the regulation of cell wall integrity, 2 chitin synthases (*chs4* and *chs9*), 2 GPI-anchored cell wall proteins (*but2* and *ecm33*), β-1,3-glucan synthase catalytic submit (*fks1*), β-1,6-glucanase precursor (*bglC*), and Concanavalin A-like lectin glucanase (*lamG*). These data suggest a role for *wetA* in transcriptional mediation of those genes involved in cell wall composition and integrity to promote conidial maturation in *M. robertsii*.

### Marked roles of WetA and VosA in intracellular mannitol accumulation.

**FIG 5:**
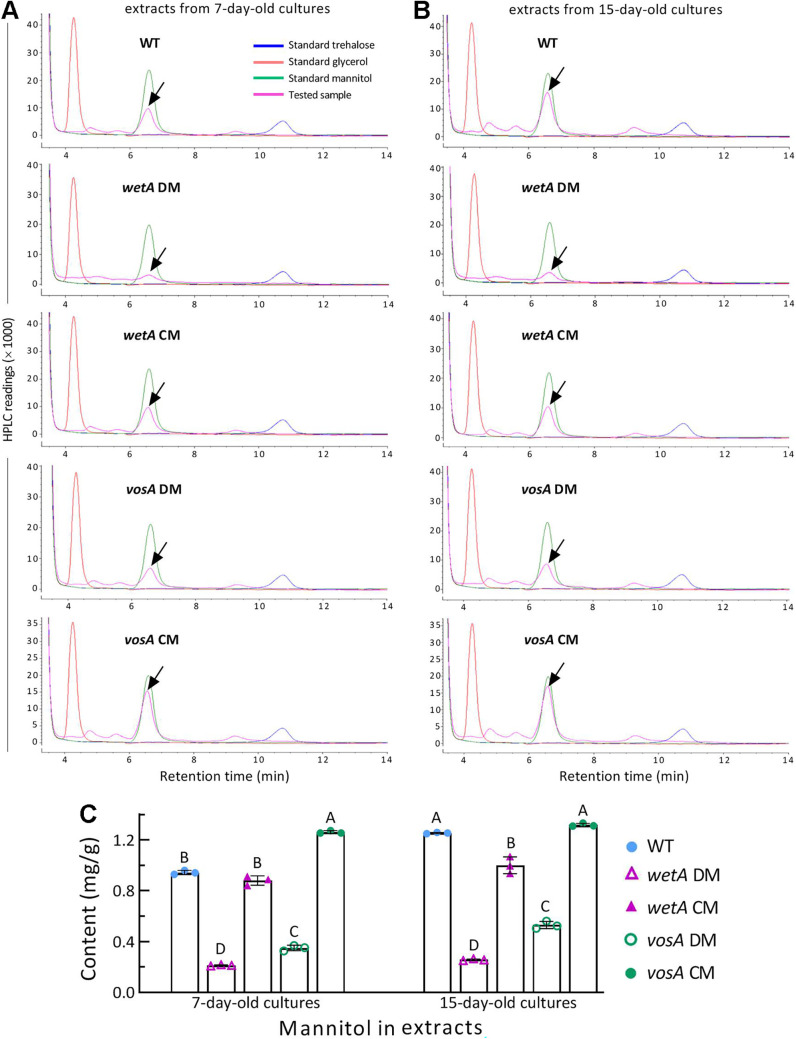
Roles of WetA and VosA in intracellular mannitol accumulation of *M. robertsii*. (A and B) HPLC curves of tested samples (arrowed) versus standard trehalose, mannitol and glycerol over retention time. Tested samples were extracted from the 7-day-old (A) and 15-day-old (B) cultures of fungal strains (DM, deletion mutant; CM, complemented mutant; WT, wild-type) grown on 1/4 SDAY at the optimal regime of 25°C and 12:12 (L:D). Note that both trehalose and glycerol were not detectable from any tested samples in comparison to their standard curves. (C) Mannitol contents quantified from the tested samples. Different uppercase letters denote a significance of *P < *0.01 in Tukey's test. Error bars: SDs of the means from 3 independent samples.

High performance liquid chromatography (HPLC) was performed to assess contents of trehalose, mannitol, and glycerol in the extracts isolated from the 7- and 15-day-old cultures of all DM and control strains. Mannitol was consistently detected in all samples of the extracts isolated from the young (Fig. 5A) or old cultures (Fig. 5B). However, neither trehalose nor glycerol was detectable from the samples of any strains compared to the standard curve of either. The mannitol contents of Δ*wetA* and Δ*vosA* were reduced respectively by 77% and 63% in the young samples, and 80% and 58% in the old samples in comparison to the control strains' counterparts (Fig. 5C). These data indicate greater role of *wetA* than of *vosA* in mannitol accumulation. The similar mannitol contents observed in the young and old cultures of either mutant implicated little role for either WetA- or VosA-reliant mannitol accumulation in conidial maturation.

### Essential versus null role of WetA versus VosA in fungal infection and virulence.

**FIG 6:**
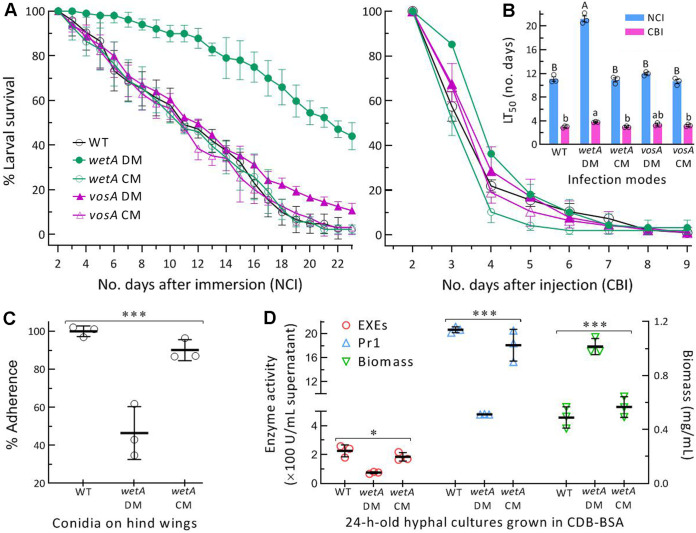
Essential versus null role of WetA versus VosA in host infection and virulence of *M. robertsii*. (A) Survival trends of G. mellonella larvae after topical application (immersion) of a 10^7^ conidia/mL suspension for normal cuticle infection (NCI) and intrahemocoel injection of ~500 conidia per larva for cuticle-bypassing infection (CBI). (B) LT_50_ estimates for tested strains against the model insect via NCI and CBI. Different lowercase or uppercase letters denote a significance of *P < *0.05 or 0.01 in Tukey's test. (C) Conidial adhesion indicated by percent ratios of post-wash counts over pre-wash counts of conidia after an 8 h incubation on locust hind wings. (D) Biomass levels and total activities (U/mL) of extracellular enzymes (ECEs) and Pr1 proteases measured from 3-day-old CDB-BSA cultures initiating by shaking a 10^4^ conidia/mL CDB-BSA at 25°C and the supernatants of those cultures. *, *P < *0.05 or ***, 0.001 in Tukey's test. Error bars: SDs of the means from 3 independent replicates.

The virulence of each strain against Galleria mellonella larvae (4th instar) was assayed by topical application (immersion) of a 10^7^ conidia/mL suspension for normal cuticle infection (NCI) or intrahemocoel injection of ~500 conidia (in 5 μL of a 10^5^ conidia/mL suspension) per larva for cuticle-bypassing infection (CBI). The means (±SD) of median lethal time (LT_50_) estimated by modeling analyses of time-survival trends (Fig. 6A) were 10.9 (±0.6) and 3.1 (±0.2) days (*n *= 9) for 3 control strains against the model insect via NCI and CBI, respectively (Fig. 6B). The Δ*vosA* mutant's LT_50_s, namely, 11.9 (±0.3) and 3.4 (±0.2) days via NCI and CBI, differed insignificantly (*P > *0.05 in Tukey's test) from the control strains' estimates. In contrast, the mean LT_50_ of Δ*wetA* was prolonged to 21.9 (±0.9) days via NCI and to 3.8 (±0.1) days via CBI. Apparently, the Δ*wetA* virulence greatly attenuated via NCI indicated an essential role for WetA in the fungal NCI.

NCI starts from conidial adherence to insect surface, followed by conidial germination and hyphal invasion into insect hemocoel under the actions of cuticle-degrading enzymes, including extracellular enzymes (ECEs), with proteolytic, chitinolytic, and lipolytic activities and subtilisin-like Pr1 family proteases (52, 53). For better insight into essential role of WetA in NCI, conidial adherence was assayed on locust hind wings, and showed a 54% reduction in Δ*wetA* compared to the WT strain (Fig. 6C). Next, total activities of ECEs and Pr1 proteases were quantified from the supernatants of submerged cultures in CDB (agar-free CDA) amended with 3% bovine serum albumin (BSA) as sole nitrogen source for enzyme induction. After the 3-day shaking incubation of a 10^4^ conidia/mL suspension in CDB-BSA, the ECEs and Pr1 activities were lowered respectively by 67% and 77% in the supernatants of the Δ*wetA* versus WT cultures, although the mutant's biomass was twice of the WT biomass in the cultures (Fig. 6D). Both enzyme activities and biomass levels differed insignificantly between the control strains. The reduced conidial adherence and the decreased ECEs/Pr1 activities accorded well with the Δ*wetA* mutant's virulence largely attenuated via NCI, reinforcing a requirement of WetA for successful NCI and insect pathogenicity of *M. robertsii*.

### Transcriptomic insight into regulatory role of WetA.

**FIG 7:**
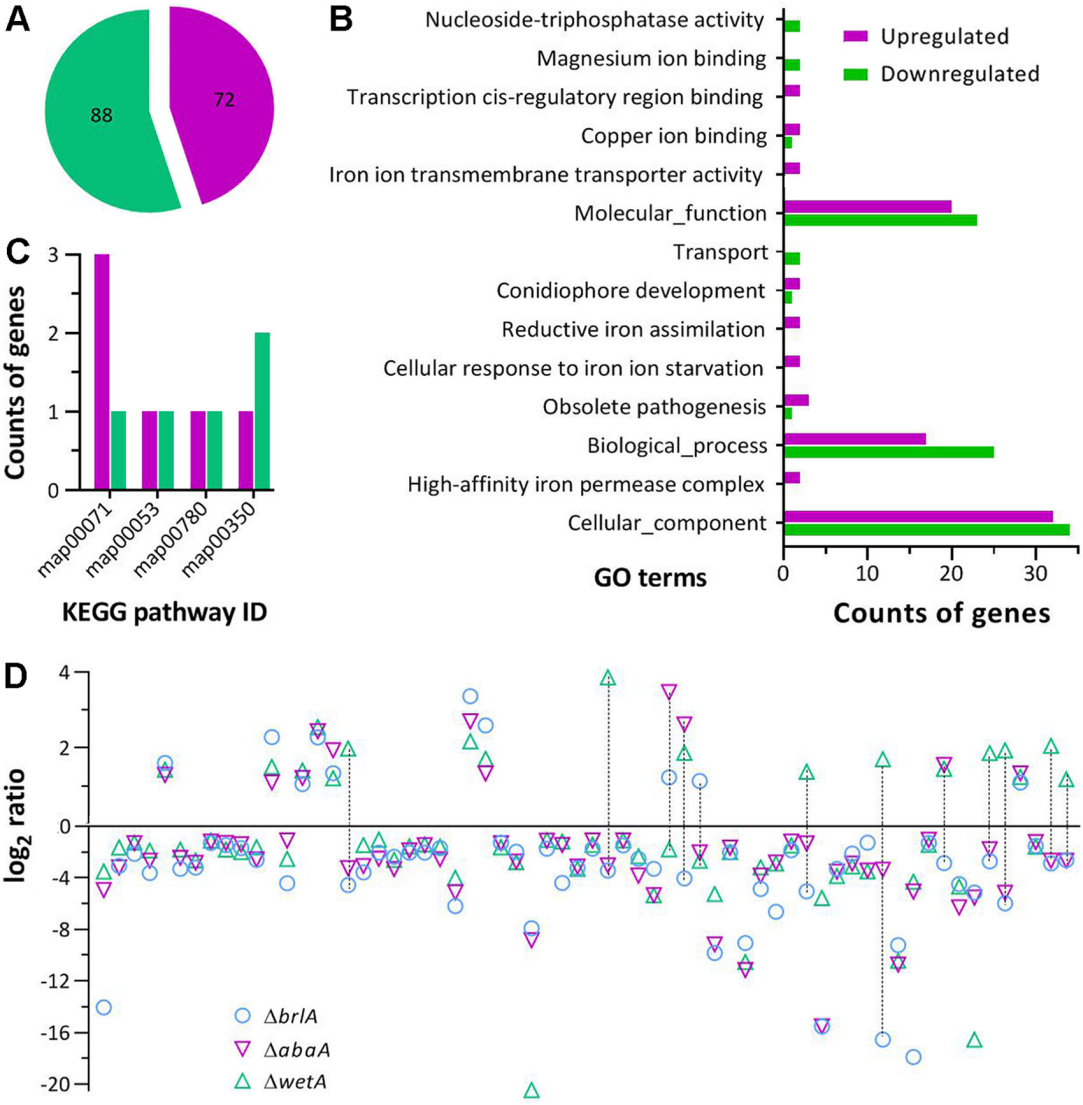
Transcriptomic analysis of differentially regulated genes in the Δ*wetA* mutant versus the WT strain of *M. robertsii*. (A) Counts of genes upregulated (log_2_ ratio ≥ 1) and downregulated (log_2_ ratio ≤ −1) at a significance of *q *< 0.05. The transcriptome was based on three 4-day-old PDA cultures (replicates) of Δ*wetA* and WT grown at 25°C and 12:12 (L:D). (B and C) Counts of differentially regulated genes significantly enriched (*P < *0.05) to GO terms and KEGG pathways (map00071, fatty acid degradation; map00053, ascorbate and aldarate metabolism; map00780, biotin metabolism; map00350, tyrosine metabolism), respectively. (D) Distribution of log_2_ ratios for all genes (detailed in Table S4) co-upregulated, co-downregulated, and differentially regulated (marked by dashed lines) in the Δ*brlA*, Δ*abaA*, and Δ*wetA* mutants of *M. robertsii.*

All phenotypes vital for asexual development, conidial quality, and host infection were compromised in the absence of *wetA* instead of *vosA*. To reveal a regulatory role of *wetA* in *M. robertsii*, a transcriptome was constructed using three 4-day-old PDA cultures (replicates) of Δ*wetA* and WT incubated at the optimal regime. Identified from the transcriptome were 160 differentially expressed genes ([DEGs]; up/down ratio: 72:88; the same meaning for ratios mentioned below) (Fig. 7A and Table S3). Up to 48 DEGs encode hypothetical or functionally unknown proteins. Gene Ontology (GO) analysis revealed 14 GO terms enriched with 177 DEGs (86:91) (Fig. 7B). The enriched GO terms included mainly cellular component (32:34), biological process (17:25), and molecular function (20:23). The remaining terms comprised only 2 to 4 DEGs *per capita*. There were only 11 DEGs enriched to 4 KEGG pathways, i.e., fatty acid degradation (3:1), ascorbate and aldarate metabolism (1:1), biotin metabolism (1:1), and tyrosine metabolism (1:2) (Fig. 7C).

Intriguingly, none of those genes analyzed via qPCR were found in the list of DEGs. Neither did the list include any known genes required for asexual development or involved in cell wall composition and hydrophobicity, providing little clue to the Δ*wetA* mutant's severe defects in aerial conidiation, conidial adhesion, and hydrophobicity. Three DEGs (2:1) enriched to the GO term conidiophore development virtually encode glucose-methanol-choline oxidoreductase (MAA_07744), C6 zinc finger protein (MAA_07622), and hypothetical protein (MAA_08878), respectively. Four DEGs (3:1) in the GO term obsolete pathogenesis encode fatty acid synthase subunit alpha reductase (MAA_01642), cell surface protein Mas1 (MAA_08289), iron permease FTR1 (MAA_08848), and hypothetical protein (MAA_01659). The high up/down ratios in the 2 GO terms are helpless for interpreting the mutant's severe defects in asexual development and NCI. Interestingly, 2 sharply downregulated subtilisin-like protease genes (log_2_ ratios: –5.35 [MAA_07742] and −16.55 [MAA_10602]) presumably involved in cuticle degradation (53) and associated with the reduced Pr1 activity, were not enriched to any GO term. On the other hand, many more DEGs enriched to the main GO terms cellular component, biological process, and molecular function could be functionally important, but not specific, for the defective phenotypes of Δ*wetA*. For example, 15 DEGs (3:15) encoding various transporters with transmembrane activity were likely involved in the activities of certain extracellular enzymes; 14 DEGs (6:8) encoding acyltransferases, methyltransferases, and phosphotransferases, which function in cellular signaling, posttranslational modification, chromatin remodeling, and transcriptional mediation (54, 55), could have exerted comprehensive effects on biological aspects of Δ*wetA*. Up to 20 DEGs (8:12) associated with carbon and nitrogen metabolisms could also have comprehensive impacts on the mutant's biology and physiology.

The Δ*wetA* transcriptome was then compared with the Δ*brlA* and Δ*abaA* transcriptomes (up/down ratios: 52:203 and 101:122, respectively), analyzed previously in *M. robertsii* (34). Surprisingly, the 3 transcriptomes comprised 64 co-dysregulated genes, including 44 co-downregulated, 8 co-upregulated, and 12 differentially regulated (Fig. 6D and Table S4). Despite few functionally known in *M. robertsii*, the co-downregulated genes could be causative of the Δ*wetA* mutant's severe defects in aerial conidiation and submerged blastospore production since both phenotypes were abolished in the absence of *brlA* or *abaA* (34). The 2 subtilisin-like protease genes, aforementioned, were also drastically downregulated in the previous Δ*brlA* and Δ*abaA* mutants incapable of hyphal invasion into insect body, suggesting their possible roles in blocking the fungal NCI in the absence of each CDP gene.

## DISCUSSION

Our data confirm the essential role of *wetA*, but no role of *vosA*, in asexual development, conidial maturation, insect pathogenicity, and cell tolerance to oxidative and cell wall stresses in *M. robertsii*. Previously, the Δ*wetA* and Δ*vosA* mutants of *B.* bassiana showed more cottony and thicker colonies, greatly (98% and 88%) reduced conidial yields, similarly increased sensitivity to NaCl-induced osmotic stress, and much more attenuated virulence via CBI than NCI, aside from moderate decreases of Δ*wetA* in conidial hydrophobicity (only ~7%), and tolerance to cell wall stress and increased sensitivity of Δ*vosA* to menadione (50). Apparently, the *wetA* orthologs in *B.* bassiana and *M. robertsii* play similar roles in asexual development but differential roles in conidial quality control and NCI. Their contributions to aerial conidiation are much greater than those reported with a 35% decrease in conidial yield of F. graminearum Δ*wetA* (46), and little effect on *Penicillium* Δ*wetA* mutants' conidial yields (47, 48). In *M. robertsii*, WetA was more involved in hydrophobin assembly onto conidial surfaces, which determine conidial hydrophobicity and adhesion essential for initial NCI (14), and also in the secretion of cuticle-degrading enzymes crucial for successful NCI (52, 53). Thus, the Δ*wetA* mutant's virulence was largely attenuated via NCI. Likewise, the reduced catalase and SOD activities were ascribed to the mutant's higher sensitivity to oxidative stress. For the Δ*wetA* mutant, impaired cell wall integrity was also shown by increased sensitivity to Congo red, easier cell wall lysing, and repressed expressions of *hyd2*, *hyd3*, and 10 other genes required for, or involved in, cell wall integrity. These observations suggest a pivotal role for WetA in mediating the cell wall composition and maturation of conidia in *M. robertsii*, and are close to the previous observations in the Δ*wetA* mutants of *Penicillium* spp. (47, 48) and F. graminearum (46). Notably, NCI and virulence were much more compromised in the Δ*wetA* mutant of *M. robertsii* than of *B.* bassiana (50). This is very different from unaffected virulence of phytopathogenic fungal Δ*wetA* mutants (46–48). The previous and present studies demonstrate that WetA othologs play relatively conserved roles in cell wall composition and maturation of conidia, but are functionally differential or even different in the lifecycles *in vitro* and *in vivo* of different insect and plant mycopathogens.

Unexpectedly, deletion of *vosA* resulted in little change in all examined phenotypes, indicating its dispensability for the asexual cycle and host infection of *M. robertsii*. This is very different from a significant role of its *B.* bassiana ortholog in conidiation capacity, conidial quality control, stress tolerance, and virulence (50), presenting 1 more big difference between the 2 insect pathogens. In Δ*vosA*, increased SOD activity could be counteracted by decreased catalase activity, leading to its null response to either H_2_O_2_ or menadione, a compound generating superoxide anions degraded by SOD into water, and H_2_O_2_ to be further decomposed by catalases (51).

Aside from spore surface structure, intracellular polyol accumulation is also a cellular event involved in conidial maturation and stress tolerance (38–40). In A. nidulans, the VelB-VosA complex was shown to mediate trehalose biogenesis and spore wall integrity (43, 44). In *B.* bassiana, single-gene deletions of mannitol-1-phosphate dehydrogenase (MPD) and mannitol dehydrogenase (MTD) resulted in reduced mannitol versus increased trehalose accumulation (56), while reduced trehalose versus increased mannitol accumulation occurred when trehalose synthesis was partially or completely inhibited by knockout mutation of the trehalose-6-phosphate synthase paralog TpsA, TpsB or both (57), suggesting reversed accumulation trends of the 2 small-molecule metabolites. Previously, trehalose contents in fresh and mature conidia from the 7- and 15-day-old cultures of *B.* bassiana Δ*wetA* and Δ*vosA* mutants grown at the optimal regime were differentially increased and decreased, respectively (50). In *M. robertsii*, trehalose content was undetectable in the extracts isolated from the young and old cultures of all DM and control strains. Instead, mannitol contents of either Δ*wetA* or Δ*vosA* decreased similarly in the young and old cultures. For the Δ*vosA* mutant, however, a decrease of ~60% in mannitol content was not influential on any examined phenotypes. The null effect of so reduced mannitol accumulation on biological aspects of *M. robertsii* agrees with the little role of mannitol as a conidial reserve carbon source in Aspergillus niger (39), and also with an inference that the metabolism of mannitol may not exist as a cycle to support its roles speculated in earlier studies (40).

Also unexpectedly, none of significantly downregulated genes in the qPCR analysis appeared in the list of identified DEGs, indicating a discrepancy between the 2 methods used in transcriptional profiling. The discrpepancy might have arisen from a 1- or 2-day difference of culture ages and more strict standards for identification of DEGs from the transcriptome. Notably, only 160 DEGs were identified in the present Δ*wetA* transcriptome. This count is very small in comparison to 5,725 DEGs (3,076:2,649) identified in A. nidulans Δ*wetA* (45), and 2,018 and 1,053 DEGs (881:1,137 and 429:624) identified from the respective Δ*wetA* cultures of F. graminearum at 6 and 12 h after conidiophore induction (46). The present and previous analyses reveal a large variation in the gene expression networks regulated by *wetA* orthologs in different ascomycetes. In *M. robertsii*, 3 functionally unknown genes enriched to the GO term conidiophore development were helpless to interpret the Δ*wetA* mutant's severe defect in asexual development. This is different from 319 conidiation-related DEGs identified from the F. graminearum Δ*wetA* less impaired in conidiation capacity (46). More small GO terms enriched provided very limited clues to deeper insight into the Δ*wetA* mutant's defects in conidial viability, hydrophobicity, adhesion, infectivity, and stress tolerance. Instead, the majority of downregulated genes in the main GO terms hint at that blocked asexual development and impaired conidial quality in Δ*wetA* could have resulted from their comprehensive roles in cellular component, biological process and molecular function. Additionally, 44 genes were co-downregulated in the Δ*brlA*, Δ*abaA* and Δ*wetA* mutants of *M. robertsii*. Since both aerial conidiation and submerged blastospore production were abolished in Δ*brlA* or Δ*abaA* (34), those co-downregulated genes, taking 27.5% of all DEGs identified from Δ*wetA*, could be important targets of WetA in *M. robertsii*. However, almost all of them remain functionally unknown like other 48 DEGs encoding hypothetical proteins in Metarhizium, warranting more studies.

Conclusively, WetA plays a relatively conserved role in regulating aerial conidiation and conidial maturation in *M. robertsii* as seen in *B.* bassiana. The process of conidial maturation regulated by WetA is linked mainly to hydrophobin assembly onto conidial coat determinant to conidial hydrophobicity and adhesion required for initial NCI, but not related to intracellular polyol accumulation. WetA is also involved in the secretion of cuticle-degrading enzymes essential for successful NCI, and, hence, plays an important role in the insect-pathogenic life cycle of *M. robertsii*. In contrast, VosA was proven to be functionally redundant in *M. roberstii*. These findings provide a scenario of WetA or VosA that is functionally differential or different from those learned in *B.* bassiana and other ascomycetes.

## MATERIALS AND METHODS

### Recognition and domain analysis of WetA and VosA in *M. robertsii*.

The amino acid sequences of WetA and VosA in A. nidulans, and of their orthologs previously characterized in *B.* bassiana (50), were used as queries to search through the genomic databases of *M. robertsii* (4) and other entomopathogenic and nonentomopathogenic ascomycetes at http://blast.ncbi.nlm.nih.gov/blast.cgi. Conserved domains were predicted from the amino acid sequences of the used queries and their orthologs located in *M. robertsii* at http://smart.embl-heidelberg.de, followed by predicting an NLS motif from each sequence with a maximal probability at https://www.novopro.cn/tools/nls-signal-prediction. All WetA and VosA orthologs found in the fungal genomes examined were clustered via phylogenetic analysis with the maximum likelihood method in the online program MEGA11 (http://www.megasoftware.net/).

### Transcriptional profiling and subcellular localization of WetA and VosA.

The qPCR analysis with paired primers (Tables S1) was carried out to assess daily expression levels of *wetA* and *vosA* in the PDA cultures of the WT strain during a 7-day incubation at the optimal regime of 25°C and 12:12 (L:D) (detailed later). For subcellular localization, the open reading frame of *wetA* or *vosA* was amplified from the WT cDNA and ligated to 5′-terminus of *gfp* at appropriate enzyme sites of the backbone plasmid pAN52-C-gfp-bar, in which C denotes a cassette (5′-EcoRI-XmaI-BamHI-PstI-HindIII-3′) driven by the endogenous promoter P*tef1* (34). The resultant vector was transformed into the WT strain via *Agrobacterium*-mediated transformation, followed by screening transgenic colonies by means of the *bar* resistance to phosphinothricin (200 μg/mL). A strong fluorescence colony selected from each transformation was incubated on PDA for conidiation at the optimal regime. The resultant conidia were incubated in SDBY (4% glucose, 1% peptone) and 1% yeast extract) for 3 days at 25°C. Hyphae from the liquid culture were washed in sterile water, stained with the nuclear dye 4′,6′-diamidine-2′-phenylindole dihydrochloride ([DAPI]; Sigma-Aldrich) of 4.16 mM, and visualized under a laser scanning confocal microscope at the excitation/emission wavelengths of 358/460 and 488/507 nm to determine subcellular localization of WetA-GFP or VosA-GFP fusion protein.

### Construction of *wetA* and *vosA* mutants.

The DM and CM strains of either *wetA* or *vosA* were constructed respectively by homologous recombination in the WT strain of p0380-5′*x*-bar-3′*x* (*x*: *wetA* or *vosA*) vectoring its *bar*-separated 5′ and 3′ coding/flanking fragments (Fig. S1A and B) and ectopic integration into an identified Δ*x* mutant of p0380-sur-*x* vectoring its full-length coding/flanking sequences by means of the mentioned method. Putative DM and CM colonies were screened respectively by the *bar* resistance to phosphinothricin (200 μg/mL) and the *sur* resistance to chlorimuron ethyl (10 μg/mL), identified via PCR analysis (Fig. S1C and D), and verified via qPCR analysis (Fig. S1E and F). The deletion vector of either target gene was constructed by amplifying its 5′ and 3′ fragments from the WT DNA and inserting into appropriate enzyme sites (BamHI*/*PstI and XhoI*/*XmaI) of linearized p0380-bar. The complement vector of each gene was generated by amplifying its full-length coding sequence and flank regions from the WT DNA and ligated into linearized p0380-sur-gateway to substitute the gateway fragment under the action of Gateway BP Clonase II Enzyme Mix (Invitrogen). Paired primers used for vector construction and detection of each target gene are listed in Table S1. The identified DM and CM strains were tested in parallel with the WT strain in the experiments, including 3 independent replicates, as follows.

### Assays for radial growth rate, stress tolerance, and spore yield and quality.

Radial growth of each fungal strain was initiated by spotting 1 μL aliquots of a 10^6^ conidia/mL suspension on the plates of PDA, SDAY, 1/4 SDAY, CDA (3% sucrose, 0.3% NaNO_3_, 0.1% K_2_HPO_4_, 0.05% KCl, 0.05% MgSO_4_, 0.001% FeSO_4_, and 1.5% agar), and CDAs amended with different carbon or inorganic/organic nitrogen sources (see Fig. 2 for details). After a 7-day incubation at the optimal regime, the diameter of each colony was measured perpendicularly to each other across the center.

To assess stress tolerance, radial growth was initiated as above on CDA alone (control) or supplemented with NaCl (0.8 M), sorbitol (1 M), H_2_O_2_ (2 mM), menadione (0.02 mM), Congo red (1 mg/mL), and calcofluor white (50 μg/mL), respectively, followed by a 7-day incubation at the optimal regime and estimation of each colony diameter as above. Relative growth inhibition [(*d*_c_−*d*_s_)/*d*_c_×100] of each strain by each chemical stress was computed using the diameters of stressed colonies (*d*_s_) and control colonies (*d*_c_).

The cultures for assessment of conidiation capacity were initiated by spreading 100 μL aliquots of a 10^7^ conidia/mL suspension on cellophane-overlaid plates (ϕ = 9 cm) of 1/4 SDAY (a medium suitable for the fungal conidiation) and incubated for 15 days at the optimal regime. From day 3 onwards, a cork borer (ϕ = 5 mm) was used to take plugs from each plate culture. Conidial yield in each plug was measured as the number of conidia per unit area (cm^2^) of plate culture as described previously (16). Biomass level was also assessed from the 7-day-old plate cultures of each strain dried at 70°C for 6 h after collection. Moreover, 50 mL aliquots of a 10^6^ conidia/mL suspension in SDBY was incubated on a shaking bed (150 rpm) for 3 days at 25°C, followed by assessment of blastospore concentration from each culture. Biomass level of each SDBY culture was assessed after the culture was collected via filtration and dried overnight at 70°C.

The quality properties of conidia collected from the 15-day-old cultures were assessed as indices of viability [GT_50_ (h) at 25°C], hydrophobicity in an aqueous-organic system, and LD_50_ (J/cm^2^) for resistance to UVB irradiation (weighted wavelength: 312 nm), as described previously (16, 17). SEM images of conidial surfaces were collected to show microstructures of hydrophobin rodlet bundles.

For deeper insight into antioxidant activity of each strain, moreover, total catalase and SOD activities (U/mg) were quantified from the protein extracts of the 3-day-old PDA cultures using Catalase Activity Assays Kit (Jiancheng Biotech) and SOD Activity assay kit (Sigma-Aldrich), following the manufacturers' guides. The impaired cell wall integrity of a given DM strain was further revealed in a cell wall lysing experiment. Briefly, the DM and control strains' cell samples (0.1 g) collected from the 3-day-old SDBY cultures were rinsed in sterile water, resuspended in 2 mL aliquots of 1.0 M NaCl containing snailase and lysing enzyme (Sigma-Aldrich) of 10 mg/mL and incubated by shaking at 37°C and 150 rpm for 3 and 6 h of cell wall lysing, followed by assessing the concentration of protoplasts released from each sample with a hemocytometer.

### Assays for contents of intracellular polyols.

Fresh samples (conidia and hyphae) of 1 g were taken from the 7- and 15-day-old cultures grown on 1/4 SDAY at the optimal regime, homogenized in liquid nitrogen, and suspended in 1 mL dd-H_2_O. The suspensions were boiled in a water bath for 6 h and centrifuged for 30 min at 1.6 × 10^4^ *g*. Intracellular trehalose, mannitol, and glycerol contents (mg/g dry mass) in each of the supernatants were determined with respect to the respective readings of standard trehalose, mannitol, and glycerol (Sigma-Aldrich) in a HPLC system described previously (58).

### Assays for fungal virulence, conidial adhesion to insect cuticle, and total activities of EXEs and Pr1 proteases.

Standardized bioassays for NCI and CBI were initiated by immersing 3 groups of 30 to 40 G. mellonella larvae per strain for 10 s in 40 mL aliquots of a 10^7^ conidia/mL suspension, and injecting 5 μL of a 10^5^ conidia/mL suspension into the hemocoel of each larva in each group, respectively. All grouped larvae were held at 25°C after inoculation and monitored for survival/mortality records at a 12 h interval until the mortality was stabilized. LT_50_ estimates were made by modeling analysis of the time-mortality trend in each group.

Conidial adhesion of each mutant required for initiation of NCI was assayed on locust (*Locusta migratoria manilensis*) hind wings with respect to the WT strain, as described previously (14, 17). Our previous protocols (59) were adopted to quantify total activities (U/mL) of cuticle-degrading ECEs and Pr1 family proteases from the supernatants of 3-day-old CDB-BSA cultures, which were prepared by shaking three 50 mL aliquots of a 10^4^ conidia/mL CDB-BSA per strain at 25°C and 150 rpm. Biomass level in each culture was also measured, as aforementioned.

### Transcriptional analysis.

Three independent PDA **c**ultures (replicates) of each strain were initiated by spreading 100 μL of a 10^7^ conidia/mL suspension per cellophane-overlaid plate and incubated for up to 7 days at the optimal regime. Total RNAs were extracted daily from the 2- to 7-day-old cultures of the WT strain or from the 4- to 6-day-old cultures of the DM and control strains using an RNAiso Plus Kit (TaKaRa), and reversely transcribed into cDNAs using a PrimeScript RT reagent kit (TaKaRa), respectively. The qPCR analysis was performed under the action of SYBR Premix *Ex Taq* (TaKaRa) to assess: (i) transcript levels of *wetA* and *vosA* from the daily WT cDNA samples with paired primers in Table S1; (ii) transcript levels of *wetA* or *vosA* in the cDNA samples derived from the 4-day-old cultures of each DM and control strains for verification of expected recombination events; and (iii) transcript levels of 3 *hyd* genes and 19 cell wall integrity-related genes in the cDNA samples derived from the 4-, 5- or 6-day-old cultures of Δ*wetA* and its control strains with paired primers in Table S2. Normalized with the fungal 18S rRNA, the 2^−ΔΔCT^ method was adopted to compute relative transcript levels of *wetA* and *vosA* in the WT cultures over the days of incubation with respect to a standard on day 2 and of other genes in the mutant strains with respect to the WT standard. A total of 1-fold transcript change was used as a standard of significant down- or upregulation.

### Transcriptomic analysis.

The *wetA*-specific transcriptome was generated in Lianchuan BioTech Co. (Hangzhou, China) based on the 4-day-old PDA cultures of the Δ*wetA* and WT strains (3 cultures per strain) grown at the optimal regime, as described previously (34). Briefly, clean tags from RNA-seq data set were mapped to *M. robertsii* genome (4). DEGs were identified at the significant levels of both log_2_ ratio (fold change) ≤ −1 or ≥ 1 and *q *< 0.05, and enriched to GO terms of 3 function categories (*P < *0.05) at http://www.geneontology.org and KEGG pathways (*P < *0.05) at http://www.genome.jp/kegg, respectively.

### Statistical analysis.

All data collected from the experiments of 3 independent replicates were subjected to one-way analysis of variance and Tukey’s honestly significant difference (HSD) test to differentiate statistical differences of phenotypic parameters between the tested DM and control strains.

### Data availability.

All data generated or analyzed during this study are included in the paper and associated supplemental files. All RNA-seq data of this study are available at the NCBI’s Gene Expression Omnibus under the accession PRJNA905185.
