# Supplementary material for: Essential Role of WetA, but No Role of VosA, in Asexual Development, Conidial Maturation and Insect Pathogenicity of Metarhizium robertsii
Source: Microbiol Spectr. 2023 Mar 14;11(2):e00070-23. doi: 10.1128/spectrum.00070-23 (PMC10100841; doi:10.1128/spectrum.00070-23)
Supplement: Supplemental file 1 — Supplemental material. Download spectrum.00070-23-s0001.pdf, PDF file, 0.8 MB [file spectrum.00070-23-s0001.pdf]

Figure S1

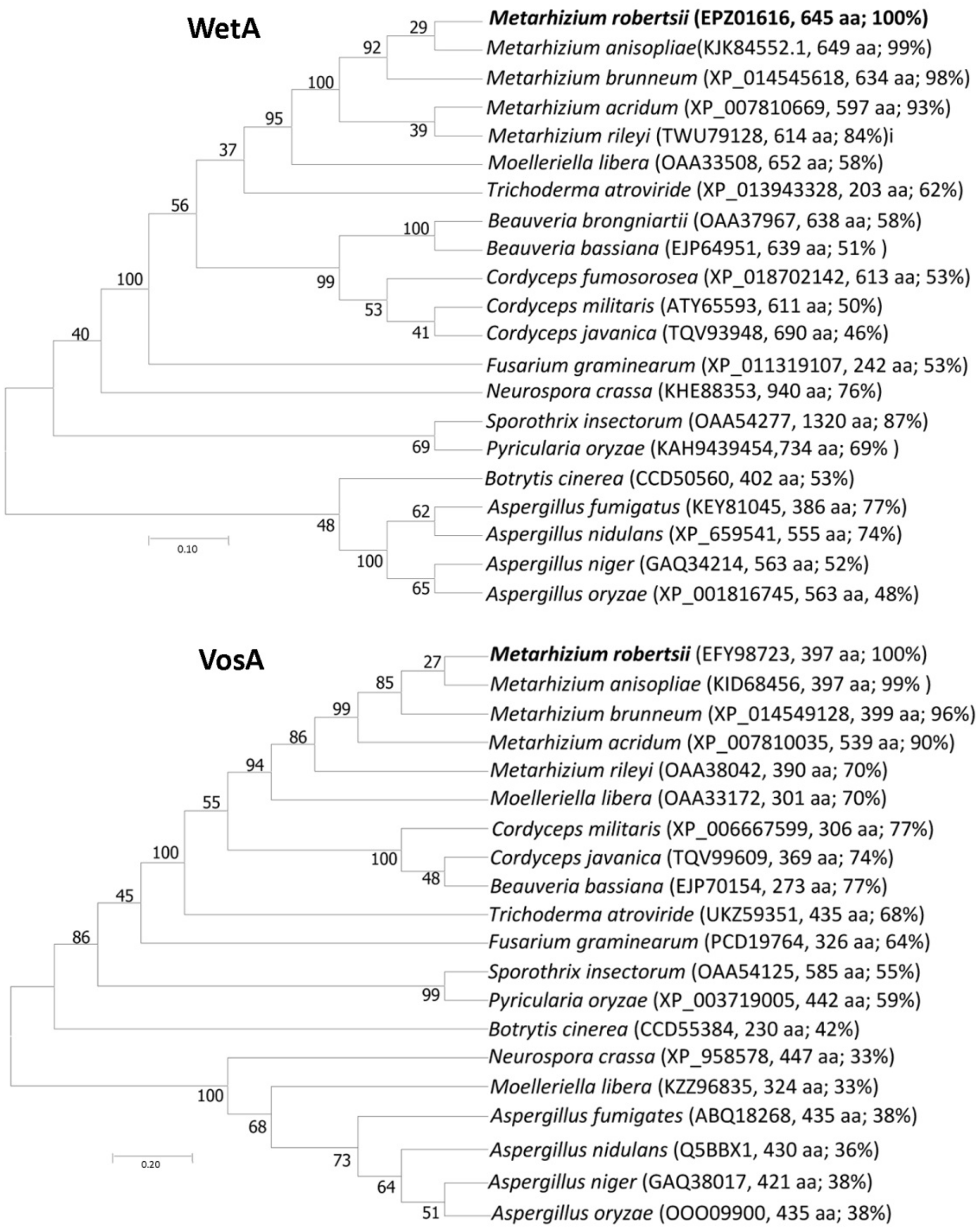

FIG S1 Phylogenetic analysis of WetA and VosA orthologs found in representative ascomycetes. Each phylogenic tree was constructed with the maximum likelihood method in the online program MEGA11 (http://www.megasoftware.net/). Bootstrap values of 1000 replications are shown at nodes. Scale bar: branch length proportional to genetic distance. The NCBI accession code and amino acid sequence length of each protein and its sequence identity to the corresponding ortholog of *M. robertsii* (in bold) are given in the parentheses following the fungal name.

Figure S2

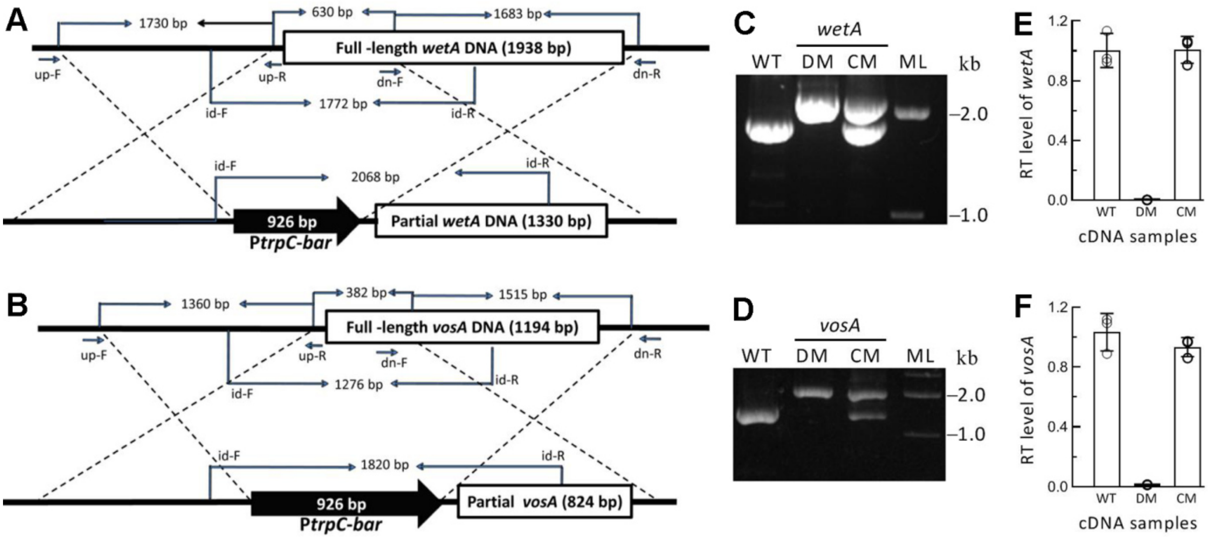

FIG S2 Construction of *wetA* and *vosA* mutants in *M. robertsii*. (**A, B**) Schematic diagrams for the disruption strategies of *wetA* and *vosA* respectively. (**C, D**) The disruption mutants (DM) and complementation mutants (CM) of *wetA* and *vosA* identified via PCR analysis with paired primers (Table S1). The genomic DNA fragments detected by PCR are 2068 and 1772 bp for the *wrtA* DM and the wild-type strain (WT), and 2343 and 1752 bp for the *vosA* DM and WT, respectively. The detected DNA fragments indicate that *wetA* and *vosA* were disrupted by deleing from the WT strain the partial promoter/coding fragment of 630 bp (1772 + 926 – 2068 = 630 bp) and 382 bp (1276 + 926 – 1820 = 382 bp) as illustrated in the diagrams, respectively. (**E, F**) Relative transcript (RT) levels of *wetA* and *vosA* in the DM/CM strains' cDNA samples analyzed via qPCR with respect to the WT standard. Error bars: standard deviation of the mean from the cDNA samples derived from three 4-day-old PDA cultures of each strain grown at the optimal regime.

Table S1. Paired primers used for manipulation and detection of *wetA* and *vosA* in *M. robertsii*.

Primers	Paired sequences (5′–3′)*	Purpose
cWetA-F/R	CACAAACACCTTCAAACCCGGGATGGCCTTTTGGACTGTTC / TGCTCACCATGTTAACGGATCCAAAAGCAAACCCCTGCTCT	Cloning *wetA* cDNA (1938 bp) for fusion to *gfp*
cVosA-F/R	CACAAACACCTTCAAACCCGGGATGTCTTTTCCATATAATGA / TGCTCACCATGTTAACGGATCCATCCCGAACAGTACGCCGCT	Cloning *vosA* cDNA (1194 bp) for fusion to *gfp*
upWetA-F/R	TAACCCGGGGATCCATGCTGCTCAGGGTCTCC / TGGGAGCTCGGTACCAAGCTTTTGTGGCTTTGGTGTTGG	Cloning *wetA* 5′-end (1730 bp) for recombination
dnWetA-F/R	CCATGGCTCGAGTCTAGAGCGACGAAATAGGAAAGCAG / CGTTAACACTAGTCAGATCTGGGTGAAAGAAGGCAGAAAA	Cloning *wetA* 3′-end (1683 bp) for recombination
upVosA-F/R	TAACCCGGGGATCCCACTGGGAAACTCTTTGCTT / TGGGAGCTCGGTACCAAGCTTTCCATACCCGAGGATTGATA	Cloning *vosA* 5′-end (1360 bp) for recombination
dnVosA-F/R	CCATGGCTCGAGTCTAGAATACAAACAAGCCGCAGCA / CGTTAACACTAGTCAGATCTGAAGCCGCCATTGAAGAG	Cloning *vosA* 3′-end (1515 bp) for recombination
flWetA-F/R	*ATCCGTCGACCTGCAGCCAAGCTT*GTTCCCAACTCCCTCCTCC / *ACACTAGTCAGATCTTCTCTAGA*GCTTCTCGTCCCGCTACAT	Cloning full-length *wetA* (2848 bp) for rescue
flVosA-F/R	*ATCCGTCGACCTGCAGCCAAGCTT*TTCTGCCTCATCCATTTACAC / *ACACTAGTCAGATCTTCTCTAGA*AGCTTTCATACGCTCTACCAC	Cloning full-length *vosA* (1848 bp) for rescue
pWetA-F/R	CATTACCCTTCAGCACCACAC / GGCGTTTGCGAACACTACAT	PCR detecting *wetA*
pVosA-F/R	TTCTGCCTCATCCATTTACAC / CACGATTTCGGTGAGTATGG	PCR detecting *vosA*
qWetA-F/R	GATCTCATCGCCCTCCCAAG / AACACTACATGGGGTGCCAG	qPCR detecting *wetA*
qVosA-F/R	TATTCCCGCCGCCATATCAC / GCTTGTGGTTGTTGTCGCAT	qPCR detecting *vosA*
18S rRNA-F/R	GAGCCAGCGAGTAATTCC/ AGCCATTCAATCGGTAGTAG	qPCR detecting 18S rRNA

*Underlined regions denote the restriction enzyme sites introduced for the fusion of *wetA* or *vosA* cDNA (*Xma*I/*Bam*HI) to *gfp* or the deletion of *wetA* or *vosA* via homologous recombination of its *bar*-separated 5′ and 3′ fragments (*Bam*HI/*Pst*I and *Xho*I/*Xma*I). Underlined and italicized regions are the recognition fragments for the gateway exchange to construct complementary plasmid.

Table S2. Paired primers used for qPCR analysis of genes involved in cell wall integrity of *M. robertsii*.

Gene	Tag locus	Annotation	Sequences (5′-3′) of paired primers
**Involved in hydrophobicity**			
hyd1	MAA_10298	Class Ⅰ hydrophobin	AGTGCGGTAACCACCAGAAG / GGGGAGCAGAGCAAGAATGT
hyd2	MAA_09713	Class Ⅱ hydrophobin	GAGATGAATTGCGACAGCGG / CAGTCTGCGAGGTGCATTTG
hyd3	MAA_01182	Hydrophobin-like protein	TGCTCCCACTTCTAACACCG / AGGGGATTTGCAGTCAAGGG
**Involved in cell wall ingridity**			
chs1	MAA_03168	Chitin synthase I	CGCATACCCATTTGCAGCTC / CTTAATCTCGCCGCAAGCAC
chs2	MAA_02740	Class Ⅱ chitin synthase	TATTTGGAGCCGTGGCACAT / TCACGGAGGTTTCGTAGTGC
chs3	MAA_02999	Chitin syntahse 3a	CTATGACCACGACCGTCTCG / CGCGTGTTGTATCGCTTCAG
chs4	MAA_01098	Chitin synthase D	GATCTCCTTGGCTACGACCG / CCCTCAAAATATGCGCTGCC
chs5	MAA_01112	Class V chitin synthase	AGGGAGGCAATAAAGGTGGC / GCTGCCGACCAAAATGGTTT
chs6	MAA_03840	Chs5-N domain containing protein	GTGGACGCCTGTGAGAAGAA / CACTTCCGCCTCTGGTTCAT
chs7	MAA_01099	Class VII chitin synthase	GGACGTTGTCGATGGTCAGT / GTTTCGTTTGCTTGCGGACT
chs8	MAA_02445	Chitin synthase chaperone-like protein	CTGCGACGCCAGAAAGAATG / ATTTGGAAGTCCCTGGCTGG
chs9	MAA_04764	Chitin synthase export chaperone	AGGACCGAGACATGACCAGA / CATGCCAATGTGGATGGCAC
fks1	MAA_04456	Beta-1,3-glucan synthase catalytic submit	TGCACCCTCATGAATGGGAC / GTACGATACAGCGTCTGCGA
bglC	MAA_00998	Beta-1,6-glucanase precursor	AGACGGCTCGGTTTGAGAAG / GCCCTTGTAGTGGTCGTTCA
lamG	MAA_09026	Concanavalin A-like lectin glucanase	ATGCGACAAGGTCACCAGAG / GTCGCCGTGGTAATACGAGT
cysZ	MAA_07401	Antigenic cell wall galactomannoprotien	CCGGAAGTGACCCCATGAAA / ACGAAGCCGAATGCTGTGTA
but2	MAA_01928	GPI anchored cell wall protein	TTGGCTGTGATGGAAGGGTC / CATATGCGCCGTTGAGGTTG
ecm33	MAA_03027	GPI anchored cell wall protein	GTCATGTCGCGGCTTCAATC / TGTTGCGGATGGTCATGTCA
smi1	MAA_01539	Cell wall assembly-coordinating protein	TAGCCCTGTTGAGCCCAATG / GCAGGCCATTCTTTCCCTCT
bck1	MAA_04583	MAP kinase kinase kinase	CTTCAACAAGCAACCGTGGG / CATGCGACTTTCGCTTCGAG
mkk1	MAA_05913	MAP kinase kinase 1	GACGGATCATCCACCGAGAC / TTCGTTCGGGTGCCATGTAA
slt2	MAA_03181	MAP kinase	TTCTGCACATCCTGGGAACC / AGTTGAACGTGGTAGGGCAG
